# HETMCL: High-Frequency Enhancement Transformer and Multi-Layer Context Learning Network for Remote Sensing Scene Classification

**DOI:** 10.3390/s25123769

**Published:** 2025-06-17

**Authors:** Haiyan Xu, Yanni Song, Gang Xu, Ke Wu, Jianguang Wen

**Affiliations:** 1 Zhejiang College of Security Technology, Wenzhou 325000, China; 20096347@zjcst.edu.cn (H.X.); 20096342@zjcst.edu.cn (G.X.); 2 Wenzhou Future City Research Institute, Wenzhou 325000, China; 3 Wenzhou Collaborative Innovation Center for Space-Borne, Airborne and Ground Monitoring Situational Awareness Technology, Wenzhou 325000, China; 4 Wenzhou Institute of Geospatial Information Technology, Wenzhou 325000, China; 5 Institute of Geophysics and Geomatics, China University of Geosciences, Wuhan 430074, China; ninis@cug.edu.cn; 6 Wenzhou Key Laboratory of Natural Disaster Remote Sensing Monitoring and Early Warning, Wenzhou 325000, China; 7 State Key Laboratory of Remote Sensing Science, Aerospace Information Research Institute, Chinese Academy of Sciences, Beijing 100101, China; wenjg@aircas.ac.cn

**Keywords:** remote sensing scene classification (RSSC), convolutional neural network (CNN), transformer

## Abstract

Remote Sensing Scene Classification (RSSC) is an important and challenging research topic. Transformer-based methods have shown encouraging performance in capturing global dependencies. However, recent studies have revealed that Transformers perform poorly in capturing high frequencies that mainly convey local information. To solve this problem, we propose a novel method based on High-Frequency Enhanced Vision Transformer and Multi-Layer Context Learning (HETMCL), which can effectively learn the comprehensive features of high-frequency and low-frequency information in visual data. First, Convolutional Neural Networks (CNNs) extract low-level spatial structures, and the Adjacent Layer Feature Fusion Module (AFFM) reduces semantic gaps between layers to enhance spatial context. Second, the High-Frequency Information Enhancement Vision Transformer (HFIE) includes a High-to-Low-Frequency Token Mixer (HLFTM), which captures high-frequency details. Finally, the Multi-Layer Context Alignment Attention (MCAA) integrates multi-layer features and contextual relationships. On UCM, AID, and NWPU datasets, HETMCL achieves state-of-the-art OA of 99.76%, 97.32%, and 95.02%, respectively, outperforming existing methods by up to 0.38%.

## 1. Introduction

**Figure 1:**
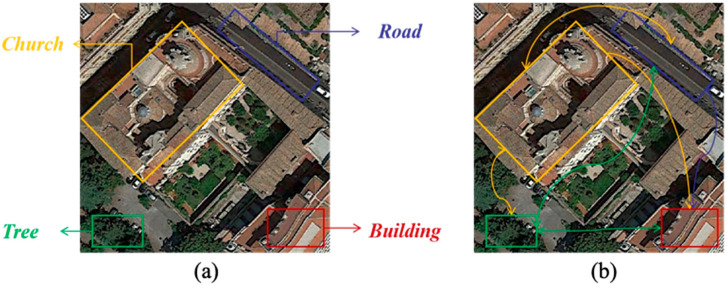
Local features and long-range contextual information selected from the “Church” class. By observation of Figure 1. (**a**), we can see that this scene contains many different types of objects, such as “Tree”, “Building”, “Road” and “Church”. If the model only focuses on and learns these local structural features, the “Church” scene may be misclassified as other scenes with similar ground objects (e.g., “Building”). Therefore, both local context information and global context information are crucial for scene classification. For example, in image (**b**), when the model can fully understand the distant dependency relationship between “Tree”, “Church”, “Road” and “Building,” the scene can be correctly classified as “Church. Therefore, leveraging the contextual semantic features across various scenes facilitates a more precise representation of scene images.

In recent years, with the development of high-resolution satellites, the spatial resolution of remote sensing has been continuously improved, and the interpretation mode has also developed from pixel level and target level to scene level. The emergence of a large number of high-resolution remote sensing image resources has made it possible to explore land surface exploration activities, and the mining and understanding of high-level semantic information of remote sensing images are crucial for understanding the current status of land use, assessing the impact of human activities, and coordinating the future development of population, economy, resources and environment, such as urban planning, natural disaster monitoring, and geographic target detection [1,2,3]. The objective of scene classification in remote sensing imagery is to accurately categorize a given image into its corresponding semantic classes based on its content. The semantic categories are mainly set according to human cognition of the geographical attributes and social functions of the features in the scene area. For instance, a remote sensing image cropped from urban areas can be classified into residential, commercial, or industrial zones. Unlike natural images, remote sensing images cover a large area and have high resolution, rich spatial information, and complex backgrounds. More importantly, the composition of the remote sensing scene is not only the accumulation of ground objects but also the image area composed of a variety of natural and socio-economic factors according to a specific spatial pattern, presenting a complex high-level semantic image area, which leads to their inter-class similarity and intra-class differences. For instance, remote sensing images often encompass diverse pixel content, whereas images of a single class frequently exhibit features from other classes. Many of these non-class-related features bear minimal relevance to the core attributes of the image class, potentially impacting the model’s classification accuracy. In Figure 1a, the predominant feature is labeled as a ‘church’, yet the pixel content includes various land cover types such as roads, trees, forests, and buildings. Moreover, remote sensing images often exhibit both intra-class similarities and inter-class differences across various image categories, which can introduce complexities in classification. These factors collectively impact classification accuracy in different classes. Therefore, improving the effectiveness of feature representation is the key to improving the scene classification performance of remote-sensing images.

Researchers have conducted several studies to reduce the impact of these problems and improve the classification accuracy of RSSC. Existing high-resolution remote sensing scene classification methods can be categorized into two groups: manual feature-based methods and deep learning-based methods. Manual feature-based methods, such as color histograms [4], directional gradient histogram (HOG) [5], and scale-invariant feature transform (SIFT) [6], have achieved relatively good results in some simple scene classification tasks. However, due to the large number and complex content of remote sensing images, designing high-performance manual descriptors is time-consuming and relies heavily on domain knowledge. In addition, handmade features can only describe scene information in a single or several aspects, which are insufficient to fully capture the complex content of remote sensing scenes and cannot meet the high-precision requirements of remote sensing scene classification.

Benefiting from the rapid development of deep learning theories, especially the emergence of Convolutional Neural Networks, deep-learning-based methods have gradually become the mainstream in the field of Remote Sensing Scene Classification (RSSC) thanks to their excellent feature extraction capabilities [7,8,9,10]. Compared with manually crafted features, the deep features learned by CNNs can describe High-Resolution Remote Sensing (HRRS) images more comprehensively. Thus, high-frequency characterization is effectively extracted. The convolution operation in CNNs is carried out through local convolutional kernels, thus endowing CNNs with strong local feature representation capabilities. However, limited by the convolution operation, each convolutional kernel mainly focuses on the local information within its receptive field, which makes it difficult to model long-range dependency relationships. For scenes with large-scale variations, global modeling capabilities are of utmost importance [11].

**Figure 2:**
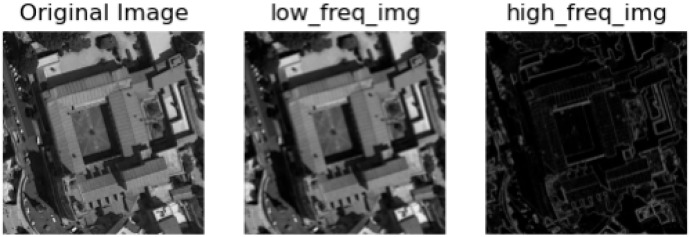
The result of the Fourier transforms selected from the “church”.

Lately, Transformer has achieved remarkable results in NLP [12,13]. Due to its multi-head self-attention (MHSA) mechanism, the Transformer exhibits significant advantages in global feature learning. As a result, Transformer is extended to specific remote sensing tasks, including scene classification [14], change detection [15], semantic segmentation [14,15,16], etc. In the domain of remote sensing, a series of Transformer-based methods have been proposed [15,16,17]. Vision Transformers can extract long-range contextual features from images and exhibit superior scene representation capabilities. Chen et al. [16] embedded a lightweight, adaptive channel compression module and a hierarchical transformer merging block into transformer architecture for acquiring rich contextual features of scene images. Sha and Li [17] inserted instance mechanisms and attention-based multilayer perceptron into the transformer for a better understanding of remote sensing scenes. In RS scene classification tasks, existing methods are typically based on the classical Vision Transformer model (ViT). ViT and its variants are able to capture low-frequency information in visual data well, mainly including the global shape and structure of a scene or object, but are not very powerful for learning high frequencies (mainly local edges and textures). This can be explained intuitively: Self-attention is the main operation in ViT used to exchange information between non-overlapping patch tokens. It is a global operation that can better capture global information (low frequency) higher than local information (high frequency). This low-frequency preference can harm the performance of ViT because (1) filling all layers of low-frequency information may deteriorate high-frequency components such as local textures, weakening the modeling ability of ViT; (2) high-frequency information is also different and can benefit many tasks, such as (fine-grained) classification. In fact, human visual systems extract basic visual features at different frequencies: low frequencies provide global information about visual stimuli, and high frequencies convey local spatial changes (e.g., local edges/textures) in the image [18,19,20,21]. As shown in Figure 2, high-frequency information includes pixels with drastic variations in image intensity (brightness/grayscale), such as local edges and textures [22]. Low-frequency information, on the other hand, includes pixels with smooth changes in image intensity, such as the global shape and structure of a scene or object, and high-frequency signals contain significant edge and contour information compared to low-frequency signals. Therefore, it is necessary to develop new ViT architectures to capture high and low frequencies in visual data.

When designing novel Transformer architectures, most researchers adopt the strategy of integrating the Transformer’s global modeling capabilities with local detail capture mechanisms. For instance, numerous hybrid CNN-Transformer frameworks have been proposed, which combine the two paradigms through either serial stacking or parallel concatenation to construct local-global feature modeling networks. Recent studies highlight the complementary advantages between CNNs and Vision Transformers (ViT), with some approaches stacking convolutional and attention layers in a sequential manner to inject local contextual information into global feature representations. However, this serial architecture inherently limits each layer to modeling only one type of dependency (local or global) at a time. Alternative approaches employ parallel designs, where convolutional and attention branches operate concurrently to learn global and local dependencies of the input data. Nevertheless, existing parallel structures often suffer from information redundancy, as they process all feature channels uniformly across each branch without adaptive weighting. To address this, the present work integrates CNN-style convolution and self-attention into a unified module while introducing multi-path branches to separately model high-frequency and low-frequency information.

To overcome the limitations of the above methods, we propose a High-Frequency Enhancement Transformer and Multi-Layer Context Learning Network for Remote Sensing Scene Classification. The main contributions of our work can be briefly summarized as follows.

1. To achieve the comprehensive integration of multi-scale information and mitigate the semantic gap between adjacent hierarchical features, the Adjacent Layer Feature Fusion Module (AFFM) is proposed. Unlike conventional multi-layer feature extraction methods, AFFM injects deep global weights into local features and then fuses them with global features, enabling the extraction of complementary global-local information from two adjacent layer features.
2. To capture high-frequency and low-frequency information in remote sensing scene images and enhance model information flow and representation capability, this paper presents a High-Frequency Enhanced Transformer (HFIE). HFIE includes a High-to-Low-Frequency Token Mixer (HLFTM). In HLFTM, HFIE employs a channel-splitting mechanism: high-frequency features are enhanced via parallel convolution/max-pooling branches, while low-frequency features are captured by downsampled self-attention. This transfers CNNs’ advantages in high-frequency capture to ViT, enhancing the latter’s spectral perception capability.
3. To model cross-level feature correlations and achieve multi-layer feature contextual aggregation, a Multi-layer Contextual Alignment Attention (MCAA) is proposed. Using three multi-layer features as keys, queries, and values and deep global semantic features as keys to guide attention matrix calculation, MCAA enables accurate focusing on critical features, facilitating a comprehensive understanding of remote sensing scenes.
4. Extensive experiments are conducted on three benchmark datasets, and encouraging results show that the proposed HETMCL is effective in RSSC.

## 2. Related Work

### 2.1. CNN–Based Methods for RSSC

In recent years, deep learning technologies, with their powerful feature extraction and pattern recognition capabilities, have sparked extensive and profound changes in the field of artificial intelligence. Among these, convolutional neural networks (CNNs), as a hallmark algorithm of deep learning, have achieved breakthroughs in numerous areas, such as image recognition and speech processing. Thanks to their unique convolutional structure and weight-sharing mechanism, CNNs have also demonstrated great potential in the field of remote sensing image analysis and processing. In 2012, Krizhevsky et al. first introduced the convolutional neural network AlexNet into the field of image classification, heralding the dominance of CNNs in classification tasks [23]. Currently, remote sensing scene classification methods based on CNNs can be roughly divided into four categories.

The first category of methods focuses on using single-depth features for remote sensing scene classification. Marmanis et al. presented a two-stage CNN scene classification structure. They employed a pre-trained CNN to extract initial features from images of different domains and then fed these features into a supervised CNN classifier. However, this method may cause low matching between the learned features and the target dataset’s features [24]. Li et al. innovatively combined transfer learning with CNNs. By fine-tuning the network to better adapt to remote-sensing data characteristics, they significantly improved the classification performance in remote-sensing scenes [25].

The second category of methods focuses on aggregating multi-layer features. Lu et al. proposed an end-to-end Feature Aggregation CNN (FACNN) and designed a novel feature encoding module. It can organically integrate and encode convolutional features from different levels, effectively fusing multi-scale features and improving the performance of remote sensing image scene classification, offering new research ideas [26]. Wang et al. proposed a multi-scale representation scene classification method based on a global-local two-stream architecture. The two-stream structure supplements key information like tiny object details, greatly improving classification accuracy [10]. He et al. combined multi-stage feature fusion with covariance pooling. By leveraging the interdependence and synergy between different features, this method transforms dispersed feature information into more discriminative representations [27]. Wang et al. proposed an Enhanced Feature Pyramid Network (EFPN). Its unique double-branch deep feature fusion module enables efficient aggregation of features at different levels, achieving good results on multiple datasets [7]. Xu et al. introduced a Graph Convolutional Network (GCN) and developed a GCN-driven Deep Feature Aggregation framework (DFAGCN) to capture global and multi-level contextual features, improving classification accuracy [9]. Li et al. proposed a new Multi-Grain and Multi-Scale Progressive Contrastive Learning Neural Network (MMPC-Net) to mine discriminative features from multi-scale and multi-grain representations and summarize discriminative knowledge between different features through a progressive contrastive learning module [28]. Additionally, many researchers have incorporated attention mechanisms into CNNs for remote sensing image scene classification. Shi et al. proposed a novel Multi-Branch Fusion Attention Network (MBFANet). By adaptively fusing two parallel modules—the efficient pooling channel attention and the efficient convolutional coordinate attention—it enhances the model’s feature extraction and generalization capabilities [29]. Hou et al. proposed an end-to-end Contextual Spatial Channel Attention Network (CSCANet) to learn multi-layer feature representations. Combining a triple loss function with a center loss function to guide model training and leveraging shallow object-level semantic information further improves classification performance [30]. Song et al. fully integrated structural features into the backbone neural network and designed improved Wavelet and Gabor feature extraction modules to effectively capture deep texture features [31]. Xia et al. proposed a Double-Branch Global-Local Attention Network (DBGA-Net) to address the issue of existing methods being unable to simultaneously focus on global and local key features [32]. Song et al. proposed a Semantic Perception Fusion Network (SAF-Net), employing spatial and channel attention to make the model focus more on key parts of input images, better capturing semantic and discriminative features [33]. Ma et al. proposed a Multi-Scale Sparse Cross-Attention Network (MSCN), emphasizing not only the effectiveness of feature learning but also the rationality of feature fusion, achieving excellent classification accuracy [34]. Zhao et al. proposed a Gradient-Guided Multi-Scale Focal Attention Network (GMFANet), introducing two modules-hierarchical multi-scale attention and gradient-guided spatial focal attention—to reduce ambiguity in model feature learning [35].

The third methods combine CNN with other technologies to further enhance classification performance. Ye et al. integrated an incremental learning mechanism to boost CNN’s classification performance and used a dynamic structure-based approach to strengthen the model’s plasticity for new tasks [36]. Wu et al. utilized an end-to-end distillation mechanism to extract class-perceptual knowledge, effectively mitigating the impact of significant intra-class variations and inter-class similarities in remote-sensing images [37]. Zhang et al. combined CNN and a graph-matching mechanism, proposing a meta-learning framework (SGMNet) based on scene graph matching, which improved classification accuracy [38]. Wan et al. integrated CNN with neural architecture search methods and proposed an efficient multi-objective evolutionary automatic search framework (E2SCNet), achieving desirable classification results. However, despite the impressive results of CNN-based methods in scene classification, their limited ability to capture the correlation between remote-sensing information and objects in scene images has constrained the classification performance of models [39].

Although CNN-based methods have achieved impressive results in RS scene classification, their ability to capture the correlation between remote information and objects in RS scene images is limited by convolution operators, which may limit the performance of scene classification tasks.

### 2.2. Transformer–Based Methods for RSSC

By learning rich features in long sequences, Transformer establishes dependencies between features at different distances [12]. After achieving remarkable success in NLP, Transformer has drawn increasing attention from researchers in computer vision. Dosovitskiy et al. applied the Transformer architecture to image classification and proposed the Vision Transformer (ViT) model [13]. ViT splits the input image into a series of image patches, which are then used to extract effective visual features for classification by exploring the dependencies between patches. Building on ViT, Bazi et al. combined transfer learning with a Vision Transformer for scene classification. They used the original ViT model to capture contextual information in remote sensing scenes and achieved satisfactory results [14]. Wang et al. proposed a Multi-level Fusion Swin Transformer (MFST) for remote sensing image scene classification. It integrates a feature merging module and an adaptive feature compression module to enhance classification performance [40]. Hao et al. employed an Inductive Bias Swin Transformer with a cyclic regressor (IBSwin-CR). By using inductive bias shifted window multi-head self-attention, it obtains inductive bias information and long-term dependencies of attention maps [41]. Lin et al. introduced a fixed-length sequence visual model. It incorporates an advanced attention encoder with an invertible residual network structure and adopts self-supervised learning to further improve classification accuracy [42].

The Vision Transformer (ViT) architecture has shown excellent performance due to its strong feature representation ability. However, it still has significant limitations. On the one hand, it requires a large number of iterative training processes to achieve satisfactory performance. This process is time-consuming and demands substantial computational resources, thereby increasing time and hardware costs. On the other hand, the attention mechanism of ViT works by calculating the correlation between each position and all other positions. While this fully connected computation method can adequately capture global dependencies in sequences, it inevitably leads to high memory consumption and computational complexity.

In light of these limitations, combining CNN with Vision Transformer to leverage the advantages of both has become an important research direction in deep learning over the past two years. Wang et al. proposed a dynamic, scalable attention model that integrates convolutional features and Vision Transformer features. It can dynamically select the model depth based on the input image size, alleviating the problem of insufficient global information extraction in a single convolutional model and reducing the computational burden of pure Transformer models. Lv et al. proposed a Spatial Channel Feature Preservation ViT (SCViT) model by improving the original ViT. Using hybrid convolutional layers and lightweight channel attention modules, it takes into account the rich geometric information in remote sensing images and the contributions of different channels to classification labels [15]. Zhang et al. integrated self-attention into ResNet in a novel way and proposed the TRS, which significantly improved classification performance and reduced dependence on convolutional operations [43]. Ma et al. proposed a Homogeneous Heterogeneous Transformation Learning (HHTL) framework that fully utilizes homogeneous and heterogeneous information in remote sensing scenes [44]. Deng et al. proposed an integrated CNN-Transformer framework, where images are processed by CNN and Transformer branches, respectively, to extract richer features [18]. Tang et al. proposed an Efficient Multi-scale Vision Transformer (EMST) that can uncover abundant multi-scale information hidden in remote sensing scenes [20]. Zhao et al. proposed a global-local dual branch structure that fully utilizes the ability of convolutional neural networks and Transformers to extract features at different scales, achieving better classification performance [45].

Recently, the proposal of Mamba has attracted a lot of attention [46]. To increase efficiency, it achieves linear complexity while guaranteeing accuracy through its competitive long-distance dependency modeling capabilities. Subsequently, the visual base model based on Manba was also well designed. Benefiting from these models, a range of Mamba-based models achieve impressive accuracy. For remote sensing image interpretation, Visual Mamba also shows good efficiency, using RSMamba for classification [47], CDMamba [48] for change detection, and several Mamba-based methods for semantic segmentation.

Most methods only consider the multi-scale features in the spatial domain, ignoring the equally abundant features in the frequency domain. Due to the characteristics of remotely sensed images, these images contain a large number of different types of interactions and connections with underlying semantic content, visually demonstrating rich spatial content such as textures, shapes, and locations. The similarity between remote sensing images is one of the main reasons for scene misclassification, and it is difficult to achieve complete accuracy by extracting features only in the spatial domain, and some easily confused features will also be extracted. Transformers address the global dependency challenge via self-attention, but their patch-based processing inherently discards High-Frequency details. Hybrid models attempt to combine CNN and Transformer, yet their uniform channel processing fails to explicitly separate High/Low-frequency features, leading to suboptimal performance on texture-rich classes.

In HETMCL, we designed a parallel structure to explore different frequency signatures and learn both global and local dependencies of inputs.

## 3. Methodology

### 3.1. Overall Structure

**Figure 3:**
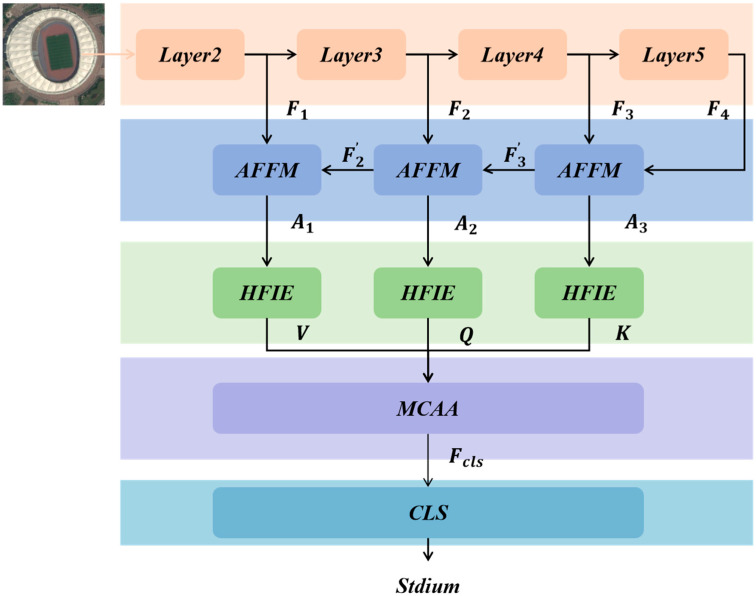
The overall framework of HETMCL.

The overall network structure of the HETMCL is shown in Figure 3. The proposed framework progressively refines features from “coarse-grained fusion” (AFFM) to “frequency-domain disentanglement” (HFIE) and finally to “fine-grained contextual alignment” (MCAA), forming a multi-dimensional feature enhancement mechanism.

AFFM is designed to narrow the semantic gap between multi-layer convolutional features and extract representative spatial multi-scale features from RS scenes. HFIE aims to establish long-range dependencies between features and generate rich semantic representations. Within this, HLFTM makes full use of shared weights and context-aware weights to extract high-frequency local representations, thus effectively understanding the RS scene within multilayer features from both the spatial and frequency dimensions. MCAA focuses on the context alignment of different layers and uses deep global semantic features as the key to guide the computation of the attention matrix. It constructs a discriminant feature representation for RS scene classification.

In the proposed architecture for RS scene analysis, the process begins with multi-layer convolutional features ([F_1_, F_2_, F_3_, F_4_]) extracted from different layers (Layer2–Layer5) of a convolutional neural network. AFFM processes these features successively, reducing the semantic gap and outputting refined features (A_1_, A_2_, A_3_). These refined features are then fed into HFIE, which, via HLFTM and other components, establishes long-range feature dependencies and extracts high-frequency local information. The outputs from HFIE are used to compute the key (K), query (Q), and value (V) matrices, which are input into MCAA. MCAA, by aligning the context across layers using the deep global semantic features as a guiding key, generates a discriminative feature representation (F_cls_) that is ultimately used for classification by the classification head (CLS). This sequential and interactive processing of features across different modules enables a more accurate and comprehensive understanding of RS scenes for classification tasks.

### 3.2. Adjacency Feature Fusion Module (AFFM)

Convolutional Neural Networks (CNNs) mainly focus on learning feature representations at the global semantic level from raw images. However, relying solely on global semantic-level features may overlook the spatial information between local objects, which is also crucial for accurate classification. When the inter-class separability is small, and the intra-class variance is large, relying only on global semantic-level features will lead to poor classification performance. To address the limitations of global high-level semantic information, we introduce low-level local features that can capture finer spatial details. To effectively fuse these features, we propose a new module: the Adjacent Layer Feature Fusion Module (AFFM).

**Figure 4:**
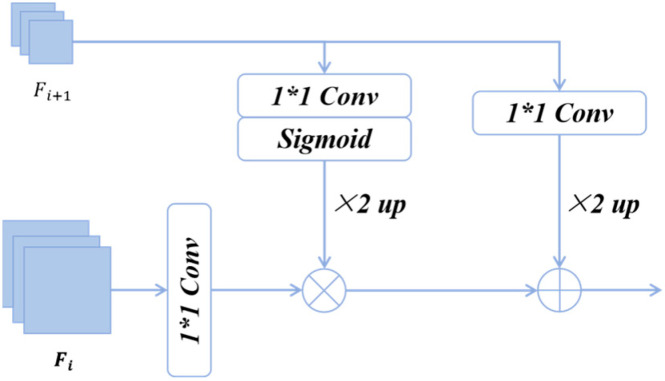
Illustration of AFFM.

In HETMCL, we select ResNet18 as the backbone network to extract features from remote-sensing images [49,50]. As a lightweight classic convolutional network, ResNet18 has fewer parameters and lower computational complexity. Moreover, it is widely used in the field of computer vision and comes with mature training and optimization schemes. Its stability and versatility have been fully verified. Using it can ensure the reproducibility of experimental results. Meanwhile, we choose four levels of features from top to bottom. Specifically, they are the outputs of the last convolutional layer of the last residual block in each stage. For the convenience of description, we represent them as [F_1_, F_2_, F_3_, F_4_]. As depicted in Figure 4, the AFFM consists of three parts: the global weights generated from deeper layers, the up-sampled global features, and the local features extracted from shallow layers. By injecting the global weights into the local features and then adding them to the global features, a set of complementary global and local information can be obtained. Specifically, the shallow feature branch undergoes convolution and batch normalization (BN) operations to generate features for fusion. The deep feature branch generates semantic weights through convolution, BN, sigmoid, and bilinear difference upsampling. These weights are multiplied with the shallow branch feature map to obtain local features enriched with global semantics. Bilinear interpolation is used to upsample the deep feature map, generating global features. Features from all three branches are projected to be the same size. Subsequently, global features are then combined with local features to create fully complementary fusion features.

The above processes can be formulated as:

(1) $$A_{i}={conv}_{1}(F_{i})*\delta ({conv}_{1}(F_{i+1}))↑+({conv}_{1}(F_{i+1}))↑$$

where i = 1, 2, 3, $F_{i}$ represents the shallow-layer features, and $F_{i+1}$ represents the deeper-layer features, ${conv}_{1}$ presents a 1 × 1 convolution and batch normalization, δ denotes the Sigmoid activation function, ↑ represents the × 2 upsampling.

### 3.3. High-Frequency Information Enhancement Vision Transformer (HFIE)

After the previous step, the features $A_{1}$, $A_{2}$, and $A_{3}$ already contain rich local information related to the RS scene. However, these features lack long-range contextual information in RS scenes. Considering the dual characteristics of remote sensing image information in the spatial and frequency dimensions, HFIE uses frequency separation technology to accurately extract high-frequency local feature representations by using the coordinated operation of shared weights and context-aware weights in the HLFTM module. This operation understands the remote sensing scene from the frequency dimension and complements the feature integration of AFFM on the spatial scale. We propose a high-frequency information enhancer (HFIE) that can effectively extract context information while capturing both high-frequency and low-frequency information in the data. HFIE combines the advantages of CNN and Transformer, enabling it to perceive global pixels through frequency-domain analysis. This approach compensates for the high-frequency texture details lost in the spatial domain due to convolution operations. Mixing high-frequency and low-frequency features enhances the model’s expressive ability and effectively captures both local and global information.

The core design of HFIE is the High-to-low-frequency Token Mixing self-attention layer (HLFTM), which adopts a dual-branched design structure.

**Figure 5:**
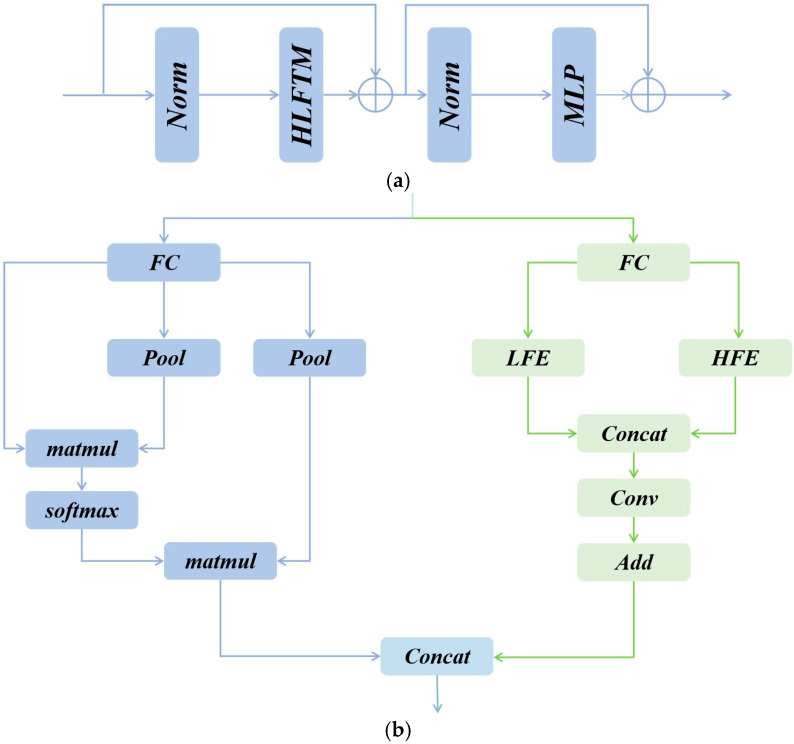
Illustration of (**a**) HFIE, (**b**) HLFTM.

As illustrated in Figure 5a, HFIE consists of a HLFTM and a multi-layer perceptron (MLP). The MLP comprises two fully connected (FC) layers and a GELU activation function.

The HFIE can be formulated as follows:

(2) $$H_{i}^{\prime }=HLFTM(LN(A_{i}))+A_{i}\;H_{i}=MLP(LN(H_{i}^{\prime }))+H_{i}^{\prime }$$

As illustrated in Figure 5b, each HLFTM is composed of a High-frequency Mixer and a Low-frequency Mixer. Here, the high-frequency Mixer is implemented by a Dual Feature Enhancer (DFE), while the low-frequency Mixer is realized through the Self-Attention operation in the Vision Transformer.

#### 3.3.1. Low-Frequency Mixer

In the low-frequency mixer, this study employs the standard multi-head self-attention mechanism to capture global information related to low frequencies. However, it should be noted that although the attention mechanism demonstrates remarkable capabilities in global representation learning and can effectively extract key feature information, the high resolution of feature maps often leads to a significant increase in computational costs. To address this issue, we downsample K and V during the attention operation. We process K and V through the pooling method, thereby effectively reducing the dimensionality of the elements. This approach ensures the effective capture of low-frequency information while reducing the high computational costs caused by high-resolution feature maps. The entire process can be expressed as:

(3) $$X_{L}=Attention(Q_{L},Pool(K_{L}),Pool(V_{L}))$$

#### 3.3.2. High-Frequency Mixer

**Figure 6:**
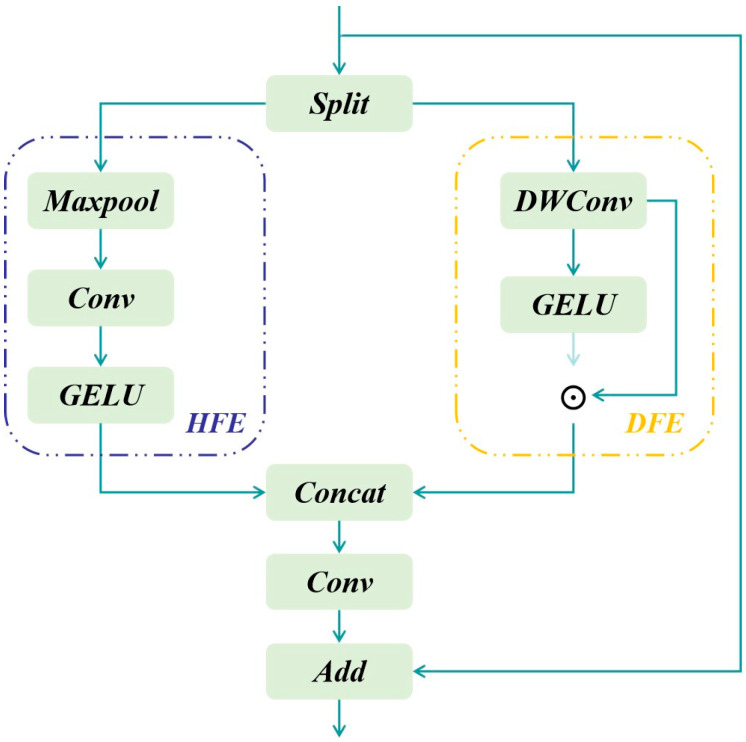
Illustration of DFE.

Although the Transformer architecture has remarkable capabilities in capturing low-frequency information, its performance in constructing high-frequency representations still has certain limitations compared to the convolutional architecture. The High-Frequency Mixer addresses the limitations of the Transformer’s global attention in HF modeling by leveraging CNN’s local sensitivity. Unlike prior hybrid models that use fixed-stride convolutions, the Dual Feature Enhancement module (DFE) employs a two-branch design (Figure 6):

In the DFE, the processing of feature information is achieved through two branches: Local Feature Extraction (LFE) and High-Frequency Detail Enhancement (HFE). The LFE branch focuses on the local details of high frequencies. The local detail extraction operation of this branch enables a more fine-grained understanding of the data and accurately grasps the subtle features of the data. The HFE branch further plays a role on this basis. Its core function is to deeply enhance the extracted high-frequency features, making the high-frequency features more discriminative.

It is worth mentioning that in order to ensure that the original information of the features is completely retained throughout the processing flow, we use the technical means of residual connections. The introduction of residual connections allows the network to still stably maintain the integrity of the original input information while learning high-frequency features, avoiding the loss of original information due to complex feature processing procedures.

As illustrated in Figure 6, in DFE, we first divide the input feature into two parts and then enter the different branches for processing:

(4) $$X_{LFE},X_{HFE}=Split(X_{in})$$

In Equation (4), $X_{LFE}$ is the input of the DFE. and $X_{HFE}$ represent the input of LFE and HFE.

Compared with the self-attention mechanism, the convolution operation has a unique inductive bias, which gives it a natural advantage in extracting local information and enables it to capture the local feature details in the data more accurately. However, the traditional convolutional feature aggregation method has certain limitations. It only relies on fixed parameter-sharing convolutional kernels for feature extraction. In this way, it is difficult for the convolution to fully consider the global context information during the processing, lacking context awareness and unable to comprehensively and flexibly deal with complex and changeable scene information.

To effectively address this issue, this study innovatively introduces a self-modulating convolution operator, aiming to organically embed context awareness into the traditional convolution operation so that the convolution can adaptively extract local representations according to different context environments. Specifically, we use the GELU (Gaussian Error Linear Units) function to generate context-aware weights and combine these weights with the depthwise convolution (DWconv). While aggregating local information more flexibly, it solves the problem of insufficient context awareness of traditional convolution. This process can be expressed as:

(5) $$X_{LFE}^{\prime }=DWconv(X_{LFE})\;X_{L}=X_{LFE}⊙GELU(X_{LFE}^{\prime })$$

This process is summarized in Equation (5), where DWConv represents a 3 × 3 Depth-Wise convolution, GELU represents the GELU activation layer, and ⊙ denotes the Hard mard product.

In the processing flow of the HFE branch, at the initial stage, a max pooling layer is used to select the key features of the high-frequency part from the input features. Then, a 1 × 1 convolution and a GELU activation function are introduced to enhance the extracted high-frequency features. Among them, the 1×1 convolution, through its ability of linear combination in the channel dimension, can flexibly adjust the number of channels of the features and optimize the feature representation; the GELU activation function introduces more nonlinear factors to the features, enhancing the expressive ability of the model, so that the high-frequency features are further strengthened, thus effectively improving the processing ability of the entire HFE branch for high-frequency information. The processing process of the entire HFE branch can be expressed in the following mathematical form:

(6) $$X_{HFE}=GELU({conv(Maxpool(X}_{HFE})))$$

In Equation (6), conv indicates a 1 × 1 convolution, Maxpool means the max-pooling layer, GELU represents the GELU activation layer.

Finally, the output results of the two branches are first concatenated and then fed into a 1 × 1 convolutional layer to thoroughly fuse the information from the two branches. To maintain the stability of training, a skip connection is introduced. The entire process can be expressed as:

(7) $$X_{H}=Conv(Concat(X_{LFE},X_{HFE}))+X_{in}$$

We adopt a simple and efficient method to fuse the outputs of the high-frequency mixer and the low-frequency mixer. The specific operation process is as follows: first, the outputs of these two mixers are concatenated in the channel dimension. In this way, the feature information of the two is integrated into the channel dimension, and the feature aggregation is initially realized. Subsequently, a fully connected layer is applied for processing in the channel dimension.

(8) $$X_{out}=FC(Concat(X_{L},X_{H}))$$

#### 3.3.3. Multi-Layer Contextual Alignment Attention (MCAA)

AFFM and HFIE have carried out in-depth mining in the spatial and frequency dimensions of features, and MCAA (Multi-Layer Context-Aware Alignment Attention) focuses on solving the problem of feature context consistency. Context differences may lead to inconsistencies in semantic understanding and information expression of the features in each layer. Therefore, how to accurately adjust the context information across these levels with obvious differences has become a key issue. Since the attention mechanism can dynamically assign weights according to the importance and correlation of different features, it provides a new approach to solving the problem of context differences.

**Figure 7:**
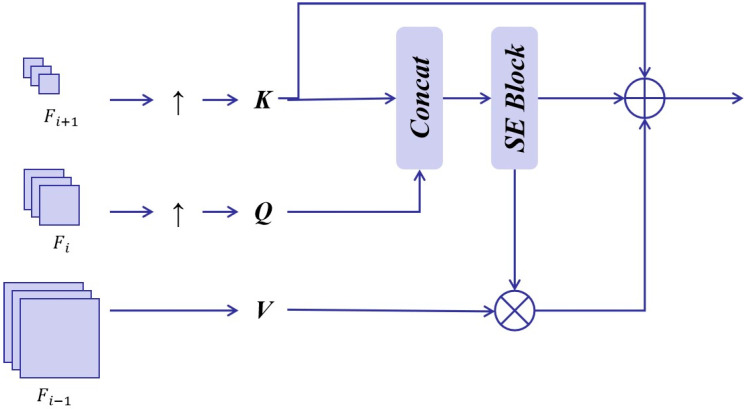
Illustration of MCAA.

Thus, in this study, we innovatively propose a Multi-layer Context Alignment Attention mechanism. The core of this method lies in skillfully using the global semantic features as the key elements to guide the calculation of the attention matrix so as to ensure accurate feature selection. As shown in Figure 7, we first perform upsampling operations on the features of three different levels to make them reach the same size so that feature selection and interaction can be carried out at a unified scale subsequently.

Deep-layer features as Key (K): Deep-layer features possess strong representational capabilities. In most neural network architectures, as the depth increases, features gradually integrate more global information and semantic abstractions. By using deep-layer features such as K, we can capture context information on a global scale, providing macroscopic and comprehensive semantic guidance for attention calculation.

Middle-layer features as Query (Q): Middle-layer features strike a balance between local and global information. Defining them as Q allows us to capture middle-level context information. When interacting with features from other layers (K and V), they can further refine and enrich the semantic connotation of the features.

Shallow-layer features as Value (V): Shallow-layer features are rich in detailed and localized information. They are responsible for providing fine-grained feature details, which are essential for tasks that require precision at the local level. In the context of attention calculation, they serve as the actual “values” that are weighted based on the interaction between Q and K, thus contributing to the final refined feature representation.

Subsequently, K and Q are organically combined, and the Squeeze-and-Excitation attention mechanism (SE) is introduced for in-depth processing. A highly discriminative global context attention matrix $A_{1}$ is generated through the adaptive adjustment of the feature channels.

(9) $$A_{1}=SE(Concat(Q,K))$$

Then, multi-layer contextual attention feature A2 is generated by aligning the local context (V) with the global context $A_{1}$:

(10) $$A_{2}=V*A_{1}$$

The final output of MCAA is to fuse the global context K with the multi-layer context participating feature $A_{2}$.

(11) $$A_{out}=K+A_{2}$$

## 4. Results

### 4.1. Datatsets

Three public RS scene datasets are used to evaluate the performance of our Method, including the UC Merced Land Use dataset (UCM) [51], the Aerial Image dataset (AID), and the NWPU-RESISC45 dataset (NWPU).

1. UCM: This dataset was published by the University of California Merced. It contains 2100 RS scene images divided into 21 scene categories. Each category consists of 100 scene images with a size of 256 × 256 pixels, and each pixel has a spatial solution of 0.3 m in the red–green–blue (RGB) color space.
2. AID: Wuhan University released this dataset. It comprises 30 scene categories and has 10,000 RS images with a size of 600 × 600 pixels. The number of images in each category varies from 220 to 420, and the spatial resolution changes from about 0.5 m to 8 m per pixel.
3. NWPU: This dataset was constructed by Northwestern Polytechnical University and has 31,500 RS scene images. These images are split into 45 scene categories, and each category contains 700 scene images with 256 × 256 pixels. The spatial resolution varies from 0.2 m to 30 m per pixel.

### 4.2. Dataset Settings and Evaluation Metrics

We repeat the experiments five times by randomly selecting training/testing samples to obtain reliable experimental results. Then, the average results and standard deviations are reported. The training–testing ratios of UCM, AID, and NWPU are set to 50%:50% and 80%:20%, 20%:80% and 50%:50%, and 10%:90% and 20%:80%, respectively. Two widely used evaluation metrics are selected to assess the classification performance, including overall accuracy (OA) and confusion matrix (CM). OA is defined as the number of correctly classified images divided by the total number of testing images, reflecting the overall performance of a classification model. CM is used to analyze the detailed classification errors and confusion degrees between different scene classes. In CM, each row/column denotes the true/predicted class.

### 4.3. Experimental Settings

All experiments were performed using Pytorch on a [52] workstation equipped with a GeForce 428 RTX 3090 graphics card and 24GB of RAM. HETMCL’s ResNet18 was initialized with pre-trained parameters (using the ImageNet dataset), while the rest of the model was initialized randomly [53]. In the data pre-processing phase, all input scenarios are uniformly adjusted to 224 × 224 resolution. At the same time, in order to enhance the diversity of data, the data augmentation strategies of random rotation (rotation angle range from −90° to 90°), horizontal flip and vertical flip were adopted to effectively expand the sample size of the dataset and improve the generalization ability of the model. During the training process, the Adam optimizer was used to train the model for 100 cycles. The initial learning rate is set to 0.0001, and a cosine decay learning rate scheduler with a linear warm-up is used to make the model more stable in the early stages of training. At the beginning of the training stage, the learning rate gradually increases to the initial learning rate through a linear warm-up strategy and then decays according to the cosine function law. The selection of 32 as the batch size ensures the reasonable use of video memory and enables the model to fully learn the data features during the training process. The batch size of 32 can balance the training speed and convergence effect under the limitation of video memory capacity and avoid the problem of insufficient video memory due to too large a batch or unstable model training due to too small a batch. The training conditions of different datasets are consistent to ensure that the experimental results are comparable and reliable, and the above parameter settings have been adjusted and optimized after multiple rounds of experiments to maximize the performance of the model so as to improve the reproducibility and credibility of the experiment.

### 4.4. Comparison with State-of-the-Art Methods

To comprehensively evaluate the classification performance of the proposed HETMCL, we compared our approach with several state-of-the-art models. The proposed HETMCL is a multi-scale method that includes contextual expressions. It combines the self-attention of CNN and Transformer within its framework. As a result, several comparative approaches were considered, including models focusing on multiscale, CNN, and transformer-related technologies. Detailed information on these comparison models is summarized in Table 1.

#### 4.4.1. Results on the UCM Dataset

The classification results of different methods on the UCM dataset are shown in Table 2.

The Overall Accuracy (OA) of ResNet18 on the UCM dataset is 97.43%. Compared with other traditional CNN models, such as CaffeNet and GoogleNet, ResNet18 has obvious advantages. Some methods focusing on multi-scale characteristics, such as EPPN-DSE-TDFF, aggregate features at different levels to obtain more refined and discriminative feature maps. Compared with traditional CNN models, they can better capture the rich information in the data and show better performance in the experimental results, which proves the effectiveness of the multi-scale feature processing strategy in improving the model performance. In addition, the Transformer-based methods have also achieved excellent results on the UCM dataset. This is mainly attributed to the self-attention mechanism of the Transformer, which can break through the limitations of local perception of traditional CNNs, correlate and integrate data features from a global perspective so as to excavate deep semantic information and demonstrate strong adaptability and processing ability when dealing with complex data feature relationships.

It is worth noting that the HETMCL method we proposed has achieved the current state-of-the-art performance on the UCM dataset. Specifically, when the training rate is 50%, comparing HETMCL with the best-performing multi-scale-based method (MF2CNet) and Transformer-based method (SCViT), HETMCL has achieved improvements of 0.38% and 0.24%, respectively, in terms of Overall Accuracy (OA). When the training rate is adjusted to 80%, the OA of HETMCL is comparable to that of the best-performing multi-scale-based method (CGINet), and compared with EMTCAL, HETMCL has achieved an improvement of 0.19% in OA. This series of comparison results clearly shows that regardless of the setting of the training rate, HETMCL demonstrates outstanding performance advantages.

#### 4.4.2. Results on the AID Dataset

The comparative accuracy of the proposed HETMCL comparison method on the AID dataset is shown in Table 3.

Among the numerous methods participating in the comparison, HETMCL has demonstrated outstanding performance advantages and achieved the best level in terms of accuracy. When the training rate is set at 20%, the accuracy of HETMCL can reach an impressive 95.91%. Compared with the second-best method (HHTL), its overall accuracy has significantly increased by 0.29%. This result clearly shows that under the condition of a relatively low training rate, HETMCL can learn key feature information by virtue of its unique architecture design and processing mechanism and then achieve more accurate classification.

When the training rate is adjusted to 50%, by comparing HETMCL with the best multi-scale-based method (MGSNet) and the Transformer-based method (Swin-T), respectively, it can be found that HETMCL has a more remarkable performance in terms of overall accuracy (OA). The improvement in OA of HETMCL has reached 0.3% and 0.4%, respectively. This means that under this training proportion, HETMCL can more fully explore the potential features in the data. Through a comprehensive analysis of the overall experimental results, it can be seen that our HETMCL has unique advantages. HETMCL focuses on the extraction of spatial frequency features and can comprehensively and deeply explore the multi-scale and context information contained in remote sensing scene images, enabling a more comprehensive and thorough understanding of remote sensing scenes.

#### 4.4.3. Results on the NWPU Dataset

The comparative accuracy of the proposed HETMCL comparison method on the NWPU dataset is shown in Table 4.

When the training rate is set at 10%, the HETMCL we proposed has demonstrated excellent performance, achieving an accuracy as high as 92.54%, which is comparable to the performance of SCViT, the best-performing method under this condition. This result fully reflects that even when the training data is relatively scarce, HETMCL can still, by virtue of its unique architecture and algorithm mechanism, learn key feature information from the limited data, thus achieving a high classification accuracy and having a good ability to deal with various scenarios with different amounts of data. When the amount of training data is increased to 20%, the advantages of HETMCL are further highlighted. It has achieved the best classification accuracy of 97.02%, and its overall accuracy improvement has reached 0.36%. This means that as the amount of training data increases, HETMCL can make full use of these data, deeply explore the feature relationships contained therein, and then classify the data more accurately, further improving its own performance.

Overall, HETMCL has demonstrated outstanding performance on the NWPU dataset under different training proportions and different amounts of data. This indicates that the proposed method does not just perform well on specific and relatively simple datasets. Instead, in a broader, more complex and data-rich context like the NWPU dataset, it can still exhibit strong generalization ability and stably and efficiently handle diverse data features and complex task scenarios.

The specific reasons can be summarized as follows: Firstly, the proposed HETMCL can excavate the local information and context information in the remote sensing image scenes to generate discriminative feature representations, showing stronger feature processing and classification capabilities. On the other hand, HETMCL can capture the high-frequency and low-frequency information in the remote sensing scene images, enhancing the information flow and expressive ability of the model so as to fully interpret the content of the remote sensing image scenes.

**Figure 8:**
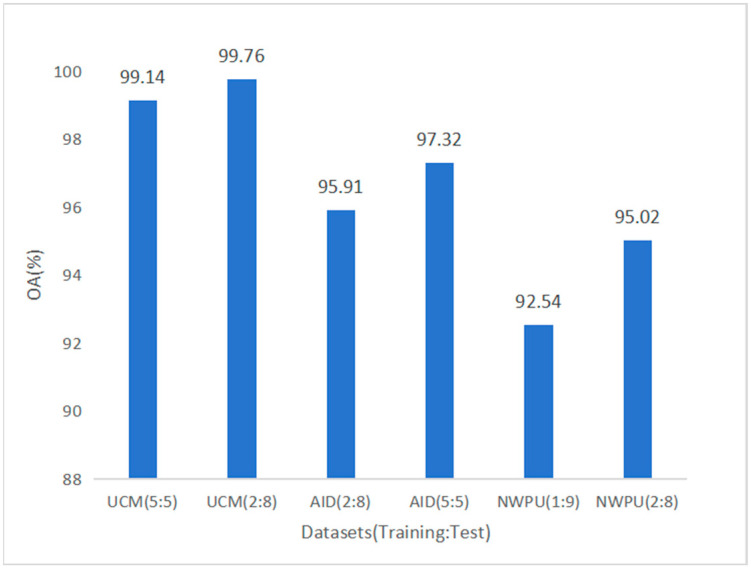
OA values of HETMCL on three datasets with different training ratios.

Figure 8 shows the OA values of HETMCL on three datasets with different training ratios. From the perspective of model learning, more training data gives the model the opportunity to learn a wider range of data features and patterns and dig deeper into the internal rules of the data, thereby improving accuracy. From the perspective of data utilization, more training data can dilute the impact of individual bias data so that the model can be trained based on more representative data and improve accuracy.

### 4.5. Confusion Matrix Analysis

To visualize the classification performance of HETMCL across different training ratios in three datasets, we generated six CM visualizations. CM can help us get a clearer view of the performance of each category. Each column represents predicted labels, and each row represents actual labels. Diagonal cells show the proportion of correctly classified samples, while off-diagonal cells represent misclassifications.

**Figure 9:**
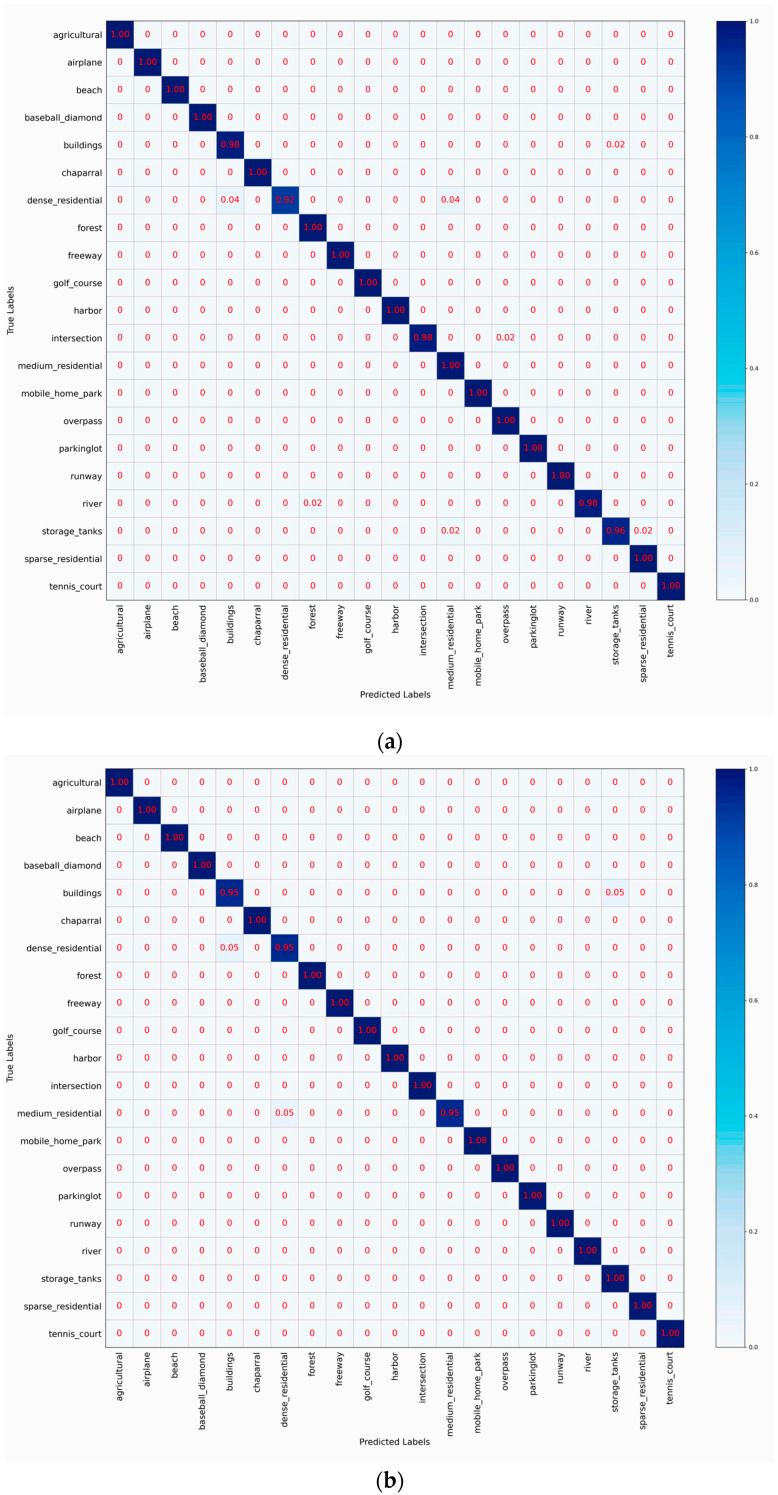
CM when 50% and 80% of the data in UCM are used for training.(**a**) 50%, (**b**) 80%.

Figure 9 shows the confusion matrices of HETMCL in the UCM data set at 50% and 80% training ratios, respectively. At 50% training ratio, our HETMCLN achieves amazing accuracy (≥95%) across most scene categories in the UCM dataset, with many even reaching 100%. With the exception of the ‘dense residential’ and ‘storage tanks’, HETMCL has a classification accuracy of more than 98% for all other scene categories. These inaccuracies can be caused by the visual similarity of the features. Specifically, ‘dense Residential’ is incorrectly classified as ‘medium residential’ and ’sparse residential’. This is because of their similar architectural styles, as well as misclassification caused by the angle and size of aerial photography. However, when the proportion of training samples increases to 80%, the classification accuracy of sparse and dense dwellings reaches 100%. This fully verifies the effectiveness of our method in solving the problem of high similarity between classes. At an 80% training ratio, only one scene class exhibits an accuracy slightly below 100%, affirming the model’s overall robust performance. The misclassifications occasionally occur between ‘medium residential’ and ‘dense residential’.

**Figure 10:**
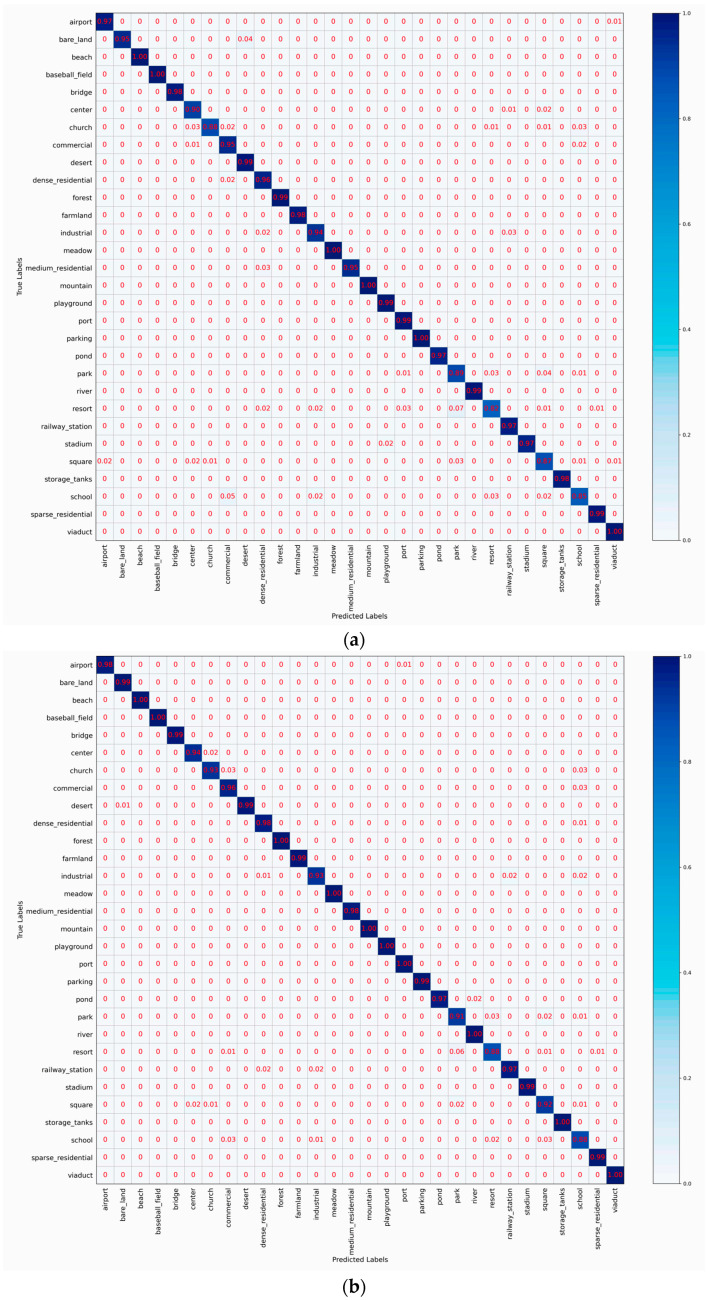
CM when 20% and 50% of the data in AID are used for training. (**a**) 20%, (**b**) 50%.

**Figure 11:**
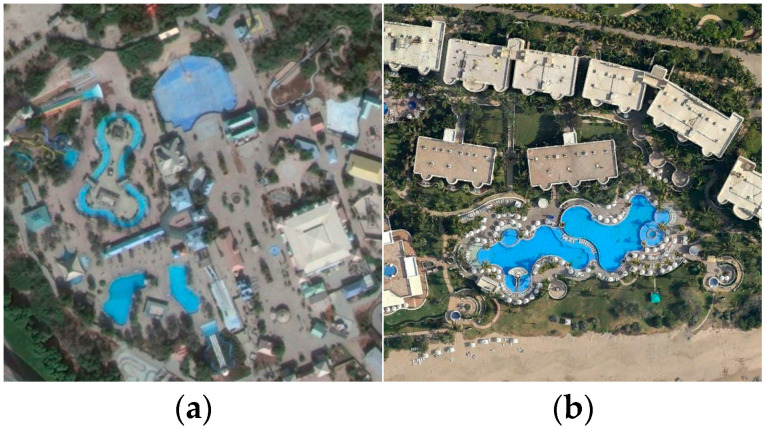
Samples from park and resort scene categories of the AID dataset: (**a**) park, (**b**)resort.

Figure 10 shows the HETMCL’s confusion matrices on the AID dataset at 20% and 50% training ratios. When the proportion of training samples is 20%, 25 out of 30 scene categories have a classification accuracy higher than 90%. Among them, the resort with the lowest classification accuracy is largely misclassified as a park. As shown in Figure 11, we can intuitively observe that Park and Resort have similar spatial layouts and architectural styles, so it is easy to lead to misclassification of models when processing related samples. However, due to the addition of the adjacency feature fusion module and the multi-layer context alignment attention module to improve the ability to extract discriminant features, our method can accurately identify other scenarios with small differences between classes, such as ‘dense residential’, ‘sparse residential’ and ’sparse residential’, with a classification accuracy of 96%, 95% and 99%, respectively. When the proportion of training samples is 50%, the classification accuracy of all 30 scene categories is not less than 90%. Among them, when the proportion of training samples is 20%, the classification accuracy of ‘church’, ‘park’ and ‘school’ with poor performance has also improved significantly in the current experiment.

**Figure 12:**
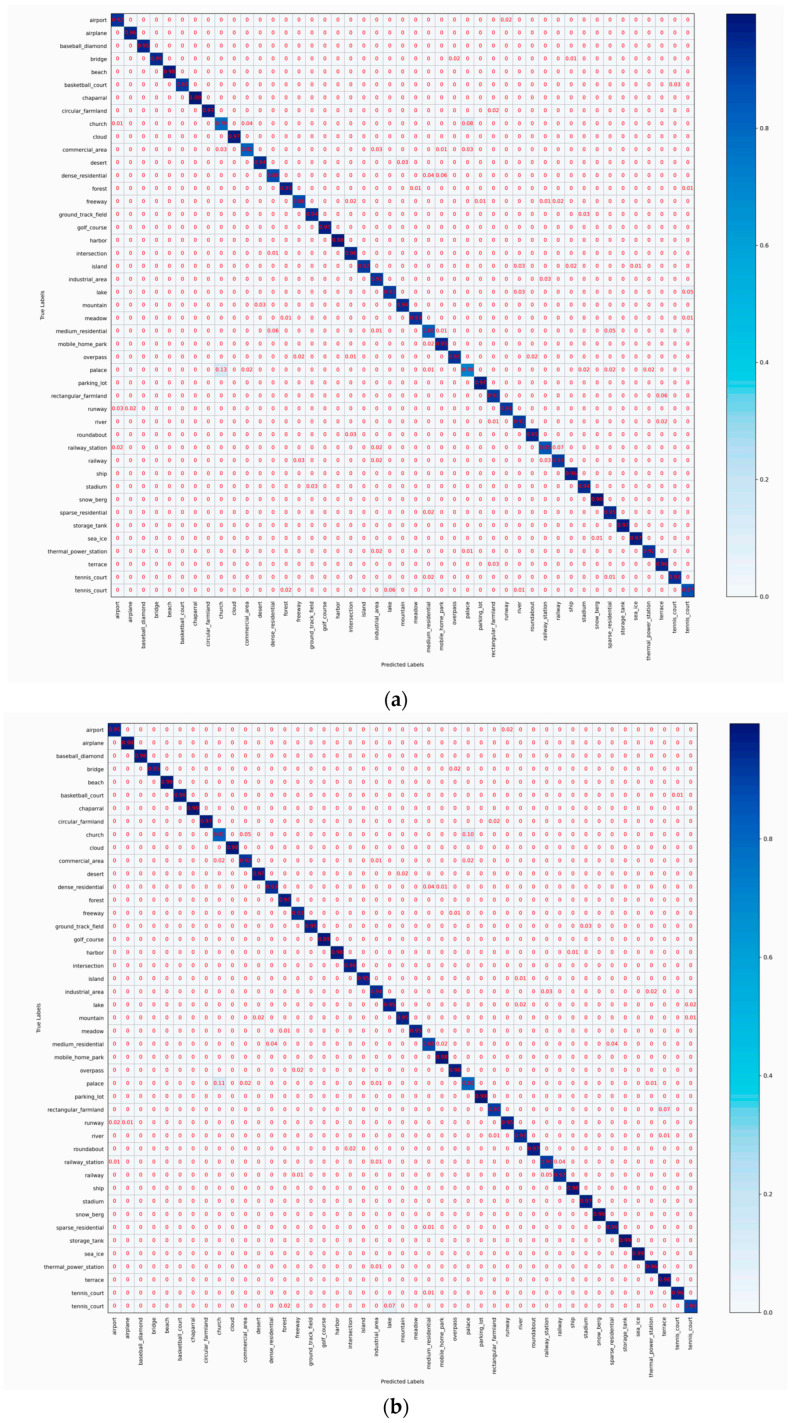
CM when 20% and 50% of the data in NWPU are used for training: (**a** )10%, (**b**) 20%.

**Figure 13:**
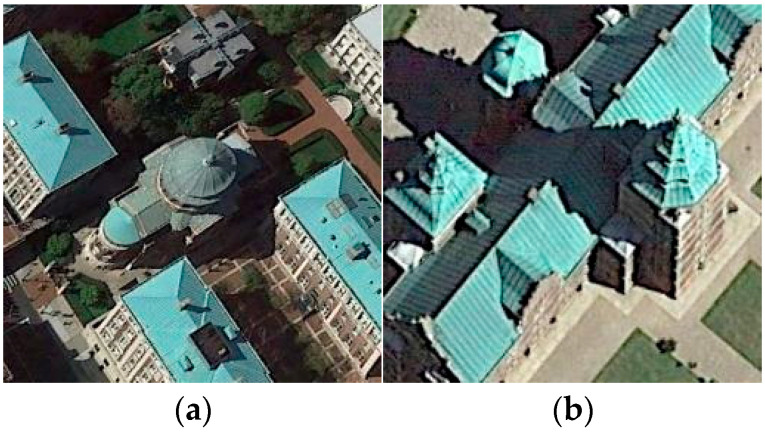
Samples from park and resort scene categories of the NWPU dataset.: (**a**) church, (**b**)palace.

Figure 12 shows the confusion matrices of HETMCL in the NWPU data set at training ratios of 10% and 20%. The NWPU data set is the most complex and largest remote sensing data set among the three data sets. Among the 45 scene categories, when the training ratio is 10% and 20%, the number of classes with classification precision above 90% is 37 and 39, respectively. In particular, both categories of church and palace had the lowest accuracy rates in both sets of experiments. And, for the most part, the ‘churches’ are wrongly classified as ‘palace’ and ‘resort’ are wrongly classified as ‘church’ to the greatest extent. As shown in Figure 13, this is because the ’church’ and ’palace’ share similar architectural styles and have some commonalities in structural and design elements, making it difficult for the model to accurately distinguish between the two when dealing with these commonalities. Furthermore, although there is a commonality problem with a large intraclass diversity in categories such as ‘airport’, the proposed method also achieves satisfactory classification accuracy. When the training ratio is 10% and 20%, the overall precision of the ‘airport’ is 92% and 95%, respectively. This is because our method guides network learning by modeling the contextual correlation between features of different scenes, so it can also solve the problem of in-class variation well in the face of more complex remote sensing datasets.

## 5. Discussion

In order to comprehensively explore the specific roles played by the proposed HETMCL as a whole and its different internal modules within the framework, a series of ablation experiments were carried out. By gradually removing certain modules from the model or changing the settings of some modules and observing the changes in the model’s performance, it is possible to clearly and intuitively analyze the contribution of each module to the overall performance and thus determine the effectiveness of each module within the entire framework. All ablation experiments were conducted on the AID dataset, with the training rates set at 20% and 50%.

### 5.1. Effectiveness of AFFM

As described in Section 2, our AFFM is designed to efficiently extract complementary multi-layered features from pretrained ResNet18. In total, the following three models were built for comparison:

(1) Net–0: HETMCL without AFFM: The AFFM is removed (features are upsampled to the same size, with simple additions).
(2) Net–1: HETMCL with FPN: The AFFM is replaced by FPN (without the global weight injecting operation).
(3) Net–2: HETMCL with AFFM.

The results are shown in Table 5. The results show that both the Adjacent Layer Feature Fusion Module (AFFM) and the Feature Pyramid Network structure (FPN) have a positive impact on the classification performance. Among several network settings, Net-2 achieves better performance than Net-1 and Net-0, and Net-0 has the worst result. The root cause of Net-0’s poor performance lies in the aliasing effect caused by the upsampling operation. The aliasing effect refers to the phenomena of feature repetition, redundancy, or mutual interference that may occur during upsampling. The upsampling operation itself is aimed at changing the size of the feature map to meet the requirements of the feature scale in the subsequent processing stage. However, in this process, there inevitably emerges the problem that it is difficult to align the high-level semantic features with the shallow-level positioning detail features. High-level semantic features often contain more abstract and generalized information, which represents a high-level understanding of the overall content of the image at the semantic level, while the shallow-level positioning detail features focus on recording the local and detailed visual information in the image, such as edges, textures, etc.

Compared with Net-0, Net-1 has a better effect. After obtaining the additive features, FPN will use a 3 × 3 convolution to fuse the generated features. The 3 × 3 convolution plays a key role here. By re-combining and optimizing the features, it eliminates the overlapping effect brought about by the upsampling process and provides higher-quality and more relevant features for the subsequent classification operation.

In all cases, Net-2 performs the best. This demonstrates the effectiveness of the global weight injection operation adopted by AFFM in extracting and integrating multi-layer features. Specifically, the global weight injection operation can consider the internal connections and respective importance degrees of features at different levels from the macroscopic perspective of the entire model, avoiding the unreasonable utilization of features or information loss. In this way, it not only enhances the semantic expression ability of a single feature but also makes the integrated multi-layer features have stronger semantic representativeness as a whole and can provide more discriminative features for subsequent classification.

### 5.2. Effectiveness of HFIE

The HFIE is designed to perceive global pixels through frequency-domain analysis and supplement the high-frequency texture details lost in the spatial domain due to the convolution operation. To verify the effectiveness of the HFIE, we compared the performance of models that only use the high-frequency mixer, the low-frequency mixer, the global branch only, and the complete HFIE. The following networks were designed:

(1) Net–0: HETMCL without HFIE: The HFIE is removed.
(2) Net–1: HETMCL without HFIE Low–frequency Mixer
(3) Net–2: HETMCL without HFIE High–frequency Mixer
(4) Net–3: HETMCL with full HFIE

The results are depicted in Table 6. Among these three networks, Net-0 has the weakest performance. After introducing the high-frequency mixer and low-frequency mixer proposed in this paper, Net-1 and Net-2 outperform Net-0 in all scenarios. This phenomenon fully demonstrates the positive roles played by these two mixer branches. The low-frequency mixer has a global receptive field, which can break through the limitations of the local scope and effectively capture the global low-frequency information from a global scale. It models the global context by calculating global self-attention, enabling the model to focus on the correlations between features, thus excavating the overall semantic and structural features.

The high-frequency mixer plays another key role in supplementing the lost high-frequency texture details. During the process of calculating the global self-attention, the operation of dividing the image into image patches will inevitably lead to the loss of some high-frequency texture details, and the high-frequency mixer can specifically make up for this deficiency. It is good at capturing texture and shape features. Supplementing the high-frequency texture details makes the feature information of the model more complete and further improves the classification accuracy.

Net-3 achieves the best results in all cases, and the main reason is that it realizes the effective fusion of high-frequency and low-frequency information. The detailed features, such as textures and shapes, that the high-frequency mixer is good at capturing form a good complementary relationship with the global modeling carried out by the low-frequency mixer. The low-frequency mixer grasps the global information from a macroscopic perspective and constructs the overall semantic and structural framework; the high-frequency mixer supplements the detailed features from a microscopic perspective, making the overall feature description more refined. Moreover, this collaborative method skillfully takes advantage of the sensitivity of CNNs to local features and the ability of Transformers to capture long-range dependencies. The advantages of the two are synergistically exerted through the fusion of high-frequency and low-frequency information, focusing the model’s attention on the important areas where the scene objects are located. Therefore, Net-3 can exhibit the best classification performance in various scenarios.

### 5.3. Effectiveness of DFE

The Dual Feature Enhancement module (DFE) enriches the feature information through two branches: Local Feature Extraction (LFE) and High-Frequency Detail Enhancement (HFE). To verify the effectiveness of the DFE, we compared the performance of different models that only use LFE, HFE and the complete DFE. The following networks were designed:

(1) Net–0: DFE without LFE.
(2) Net–1: DFE without HFE.
(3) Net–2: DFE.

As shown in Table 7, we can draw the following conclusions: Firstly, regarding the limitations of a single branch, neither HFE nor LFE alone is sufficient to learn excellent representations. Although LFE focuses on extracting local features, in some complex tasks, relying solely on local features makes it difficult to comprehensively capture the key information of the data. Similarly, although HFE can enhance high-frequency information and highlight the detailed parts without fine extraction and integration of local features, it is also difficult to construct a comprehensive and accurate feature representation. Secondly, the complete DFE achieves the best performance, which fully proves the effectiveness of the design concept of DFE. By combining the two branches of LFE and HFE, DFE realizes the complementarity of features at different frequencies. The local features extracted by LFE provide the model with basic, detailed information, and HFE enhances the high-frequency details through the prior high-frequency information from CNN, enabling the model to capture the features in the data more comprehensively and accurately. When only shared weights are used to extract high-frequency local information, the classification accuracy is 0.30% lower than that of the complete DFE. Similarly, when only context-aware weights are used, the result decreases by 0.05%, which indicates that the way of using weights has an important impact on the model performance. Both shared weights and context-aware weights play specific roles in DFE. When only shared weights are used to extract high-frequency local information, the model may not be able to adaptively adjust the weights according to different data features, resulting in inaccurate extraction of high-frequency local information. When only context-aware weights are used, although the context relationship of the data is considered to a certain extent, the mining of local information may not be sufficient, which also affects the model performance. This shows that in DFE, a reasonable combination of different weight settings is crucial for achieving the best performance.

### 5.4. Effectiveness of MCAA

MCAA is proposed to aggregate the contributions of multi-layer features. Ablation experiments are performed as follows:

(1) Net–0: HETMCL without MCAA: The MCAA is removed.
(2) Net–1: HETMCL with MCAA but change the input.
(3) Net–2: HETMCL with MCAA.

The performance is illustrated in Table 8.

The performance of Net-1 has slightly declined. This is mainly due to the confusion of semantic information caused by the exchange of Q (query) and V (value). In the model system based on the attention mechanism, Q, K, and V have their own specific functions and meanings. After the roles of Q and V are interchanged, the originally orderly information interaction logic is broken, making it difficult for the model to effectively process data and resulting in a decrease in performance. Net-2 has obtained the best results in all cases. Using the global context to guide the attention matrix enables the attention matrix to allocate weights more reasonably and coordinate the context information between different layers so that the model can comprehensively and effectively integrate features at different levels.

### 5.5. Analysis of HFIE

(1) Comparison with Other Architectures

In order to more fully demonstrate its performance advantages and competitiveness among similar methods, we selected multiple representative Vision Transformer architectures for comparison. These include the original Vision Transformer architecture, namely ViT [14], as well as several improved Transformer architectures that have shown certain influence in related fields, including Pyramid Vision Transformer (PVT [61]), Swin Transformer (Swin [59]), and CMT [15]. To ensure fairness, we only replaced the HFIE with ViT, PVT, Swin, and CMT, respectively, to construct new networks so as to observe the impact of the integration of different architectures on the performance of the entire model. The results are shown in Table 9.

Judging from the tabular data, all models participating in the comparison showed acceptable overall performance on multiple performance metrics. Although there are differences in calculation quantity (GFLOPs), parameter quantity (Para (M)) and inference speed (FPS), the values of the key evaluation indicators are at a high level under the conditions of Tr = 20% and Tr = 50% (both above 95), which fully verifies the powerful context information learning ability of the transformer model.

In the comparative experiments of the HETMCL model with different Transformer architectures, the data further reveals performance differences. In terms of the comprehensive performance of computational complexity, parameter count, and inference speed, HETMCL models equipped with PVT, Swin, and CMT exhibit more significant advantages compared to HETMCL using ViT. Among them, although HETMCL based on the Swin architecture has a slightly lower inference speed than the ViT architecture (50.72 FPS vs. 52.96 FPS), it still demonstrates excellent performance indicators under the test conditions of Tr = 20% and Tr = 50%, indicating that this type of architecture can better balance computational resources and model performance. It is worth emphasizing that the proposed HETMCL method achieves optimal classification performance in all comparison scenarios. Taking HETMCL integrated with the HFIE module as an example, when Tr is 20% and 50%, its classification accuracies reach 95.91 and 97.32, respectively, which are significantly higher than those of other comparative models. In the quantitative comparison with ViT, Swin-T, and CMT, although HETMCL has slightly higher GFLOPs (12.28 vs. 10.36 for Swin-T), its parameter count (20.08M) remains competitive, and the FPS (50.23) is within 3% of Swin-T (51.72). In terms of classification accuracy, a critical performance indicator, HETMCL demonstrates significant improvements. Take HETMCL integrated with the HFIE module as an example: its classification accuracy reaches 97.32 at a test ratio (Tr) of 50%, significantly outperforming other comparative models. Such performance improvements typically come at the cost of increased computational load. However, when the practical value brought by accuracy improvements surpasses the computational cost, the increase in computational load becomes justifiable. Through evaluation, the increase in model computational complexity is still within an acceptable range, which indicates that while pursuing high performance, HETMCL does not lose its practical application feasibility due to the increase in computational volume.

Compared with other methods, HETMCL innovatively introduces a High-Frequency and Low-Frequency Feature Interaction Module (HFIE). By fusing feature information of different frequencies, this module can more efficiently capture the detailed textures and global structures in remote sensing images. In contrast, PVT adopts a progressive pyramid structure to enhance multi-scale feature representation, and Swin introduces a hierarchical windowing mechanism, dividing the input image into several overlapping local windows, and applies Transformer operations within these windows. CMT replaces the self-attention mechanism with convolution. None of these models involve explicit processing of frequency features, while the HFIE module of HETMCL fills this gap and provides a brand-new idea for the design of hybrid models.

(2) The Influence of the Number of HFIE

In the High-Frequency Information Enhancement (HFIE) component, the number of HLFTM modules significantly influences model behavior. Stacking multiple HLFTMs enhances the model’s capacity to learn complex data representations through hierarchical feature abstraction. However, excessive module accumulation introduces challenges: overfitting risks escalate as redundant components may overemphasize training data noise and trivial details, undermining the model’s generalization to unseen samples. Concurrently, deep stacking of HLFTMs may induce gradient instability issues, such as vanishing or exploding gradients, during backpropagation.

**Figure 14:**
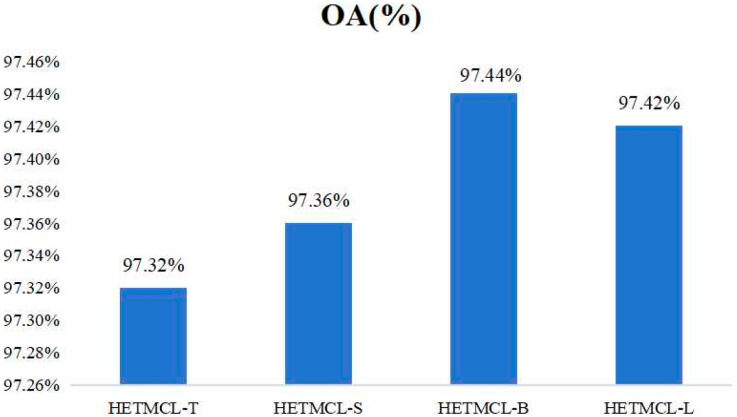
OA values of different HETMCLs.

To systematically analyze the impact of HLFTM count on model performance, we instantiated a series of HETMCL variants with varying module configurations: we used one HLFTM to construct HETMCL-T, two HLFTMs to construct HETMCL-S, three HLFTMs to construct HETMCL-B, and four HLFTMs to construct HETMCL-L. In this way, we can intuitively observe the specific change trend of the model performance as the number of HLFTMs changes. From the performance results of different models shown in Figure 14, we found that the performance of HETMCL under different parameter values k (that is, different numbers of HLFTMs) is relatively similar. This phenomenon indicates that our HETMCL is not sensitive to the selection of the parameter k; that is, the model can adapt to changes in the number of HLFTMs to a certain extent without significant fluctuations in performance. Although there is a slight upward trend in performance as the number of HLFTMs increases, considering the balance between computational complexity and performance in practical applications, an excessive number of HLFTMs will increase the consumption of computational resources and the computation time, while the performance improvement is not significant. Therefore, after a comprehensive trade-off, we finally chose to use only one HLFTM to construct HETMCL. In this way, it can not only ensure that the model has a certain performance level but also maintain a good balance in terms of computational resources and time costs.

(3) Spectral Analysis

Through performing a two-dimensional Fourier transform (2D-FFT) on visualized feature maps, the spectral distribution of features can be characterized. The 2D-FFT enables the conversion of images from the spatial domain to the frequency domain, where frequency components at distinct positions encode feature information across different scales and orientations. Specifically, the central region of the frequency spectrum represents low-frequency components corresponding to global image characteristics such as general contours and smooth regions, while peripheral regions denote high-frequency components associated with local details, including edges, textures, and fine structures.

**Figure 15:**
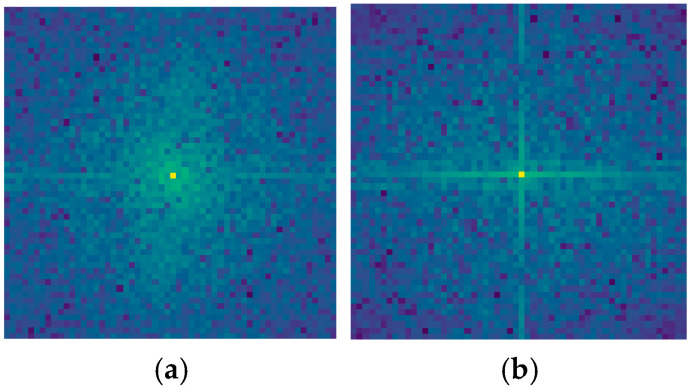
Spectral analysis of HETMCL: (**a**) only the Low-frequency Mixer, (**b**) the complete HFIE structure.

During the spectral analysis, we conducted an in-depth investigation of feature maps from three hierarchical levels of HFIE modules within HETMCL. Taking the second-level HFIE as an illustrative case, we compared two critical feature maps: one constructed using only the low-frequency mixer and the other employing the full HFIE architecture integrating both low-frequency and high-frequency mixers. The comparative results are visualized in Figure 15.

From the comparison of the frequency spectrum diagrams, it is clear that there are significant differences in the frequency components of the feature maps constructed by the two methods. The central bright spots in both diagrams represent low-frequency components. The central bright spot in Figure 15a is brighter than that in the left-hand diagram, and the surrounding green area is larger and brighter. This indicates that the low-frequency components in the corresponding feature map have stronger energy and a larger proportion, meaning that it contains more information about the global structure of the image. The high-frequency region is relatively dim, suggesting that the high-frequency components in the feature map corresponding to the spectrum of the feature map constructed only with a low-frequency mixer have relatively lower energy. The low-frequency components dominate, which means that this method performs outstandingly in obtaining the macroscopic and overall information of the image. For example, a remote-sensing image can better capture the general outline of the terrain and the overall distribution of urban areas. However, due to the relatively low energy of the high-frequency components, its ability to capture detailed information, such as the fine structure of buildings and the texture of roads in the image, is relatively limited. In Figure 15b, the high-frequency region away from the center is relatively more prominent, with brighter points distributed around. The high-frequency components in the corresponding feature map have relatively stronger energy and contain more information about details and edges. This enables it to keenly capture the detailed features in the image, such as the edge trends of rivers and the texture differences of vegetation in remote-sensing images. By combining the low-frequency and high-frequency mixers, the complete HFIE structure can extract and retain high-frequency information and performs well in capturing both the macroscopic features and local details of the image.

To analyze the proportions of each frequency, we performed a Power Spectral Density (PSD) calculation, and the results are as follows in Table 10:

This metric quantifies the relative emphasis on high-frequency details. As shown in Figure 15, the full HFIE architecture exhibits a 46% increase in EPI compared to the low-frequency-only variant, indicating enhanced retention of fine-grained features. Spectral entropy analysis further reveals that the full HFIE achieves a 12.7% higher entropy value (3.21 vs. 2.85 nats), demonstrating a more balanced distribution of information across frequency bands. Specifically, the central luminance in Figure 15a exhibits a 37.2% higher pixel intensity (measured via region-of-interest analysis) compared to Figure 15b, with the surrounding green area (indicating intermediate frequencies) expanding by 29.5% in radius and 41.8% in integrated brightness. These metrics indicate that the low-frequency mixer-only approach retains stronger energy in global structural features, accounting for 83.2±1.7% of total spectral power.

### 5.6. Visual Analysis

**Figure 16:**
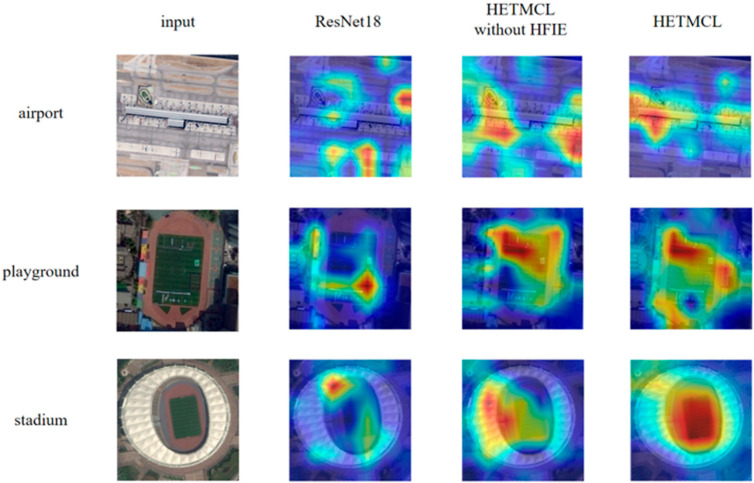
Visualizing results of HETMCL using Grad-Cam.

In this section, we use the Grad-CAM (Gradient-weighted Class Activation Mapping) technique to generate visual explanations. The core objective is to highlight the regions in the image that play a crucial role in predicting the corresponding objects. In this way, we can intuitively understand which parts of the image the model mainly focuses on when making classification decisions. In the class activation map, the shade of color represents the weight level. The darker the color, the higher the attention of the model to that region.The results are shown at Figure 16.

Target Region Recognition Accuracy: On the AID dataset, HETMCL outperforms ResNet18 in remote sensing scene classification. ResNet18 roughly identifies target areas but lacks detail, with diffuse activation (e.g., scattered across airport edges). HETMCL without HFIE focuses more on core regions (e.g., airport buildings/runways) but still has scattered activations. In contrast, full HETMCL concentrates attention tightly on classification-relevant areas, enabling precise localization.

Widely Distributed Target Recognition: For playground images, ResNet18’s activation scatters across sports fields and surroundings, indicating interference and incomplete target coverage. The HFIE-free model focuses on lawns and runways but retains edge activations. HETMCL comprehensively covers core sports areas, suppresses irrelevant edges, and extracts features more accurately for superior classification.

The visualization results reveal the internal mechanism of the model’s classification from the perspective of feature-focused regions. Subsequently, we used OA and Kappa to further demonstrate the classification performance of the model on different datasets from a quantitative perspective.The results are shown at Table 11.

On the UCM dataset, the OA and Kappa coefficients under both division ratios are extremely high, indicating that the model has very good classification accuracy and consistency on this dataset. The classification results of the algorithm have a strong consistency with the real situation, and the influence of random factors on the classification results is minimal, showing excellent algorithm performance.

On the AID dataset, both the OA and Kappa coefficients are relatively high, indicating that the classification results of the algorithm have good consistency with the real situation. As the ratio of the training set to the test set changes, the performance of the model also improves. The indicators under the (5:5) division are higher than those under the (2:8) division, suggesting that a more balanced data division is conducive to the model’s learning, thereby improving classification accuracy and consistency.

On the NWPU dataset, although the OA and Kappa are relatively lower compared to the other two datasets as a whole, the performance of the algorithm also improves as the proportion of the training set increases. The indicators under the (2:8) division are higher than those under the (1:9) division, also indicating that increasing the proportion of the training set can improve the classification performance of the model.

Combining the results of visualization and quantitative analysis, the HETMCL model demonstrates unique advantages. At the visualization level, it can accurately focus on the key features of the target area, intuitively reflecting its ability to capture core information. In terms of quantitative indicators, although its performance varies across different datasets, the overall trend is positive, highlighting the model’s adaptability to data structures.

## 6. Conclusions

In this study, we proposed a context-aware local enhanced transformer and multi-layer context learning network (HETMCL) for remote sensing scene classification. The model successfully integrates the characteristics of convolutional neural network (CNN) and transformer (Transformer) while retaining their respective advantages.

Specifically, we use ResNet18 as the backbone network to extract convolutional features of different levels from remote sensing images and then use the adjacent layer feature fusion module (AFFM) to perform adjacent fusion of these convolutional features of different levels and use high-level features to refine low-level features, effectively narrowing the semantic gap between multi-layer convolutional features. The high-frequency information-enhanced visual transformer (HFIE) fully utilizes the sensitivity of CNN to local features and the ability of the Transformer to capture long-range dependencies. Through the innovative dual-branch structure design of high-low frequency marker mixture (HLFTM), it effectively models the context of remote sensing scenes from the spatial and frequency dimensions, successfully captures the high-frequency and low-frequency information in the data, supplements the high-frequency texture details lost in the spatial domain due to convolution operations, enhances the expressiveness of the model, and enables the model to understand the scene content more comprehensively. The multi-layer contextual alignment attention (MCAA) mechanism cleverly uses the global context to coordinate the contextual information between different layers, effectively aggregating the contributions of multi-layer features, thereby further improving the model’s ability to understand and classify complex scenes. Through a large number of experiments on three widely used datasets, the results clearly show that the HETMCL model exhibits excellent performance in remote sensing scene classification tasks, and its classification accuracy is significantly better than many existing methods, which strongly proves the effectiveness and advancement of our proposed method.

Although HETMCL has achieved encouraging results, we are also aware that there is still room for further performance improvement. In future research, we plan to explore more effective ways to achieve a tighter and more efficient fusion between top-level features and aggregated multi-layer features and further optimize the model’s utilization efficiency of features at different levels. In addition, we will also actively study the application potential of other frequency domain analysis techniques (such as Fourier transform [62]) in the model, hoping to mine more hidden information in remote sensing images through deeper frequency domain analysis. In addition, we plan to conduct further research on the dual-branch remote sensing scene classification model based on CNN and Transformer. Future work will explore integrating lightweight sequence models and adaptive frequency-domain filters to further enhance efficiency and feature representation”.

## Figures and Tables

**Table 1:** Detailed Types And Publication Years Of Different Methods.

Type	Methods	Publication
CNN	CaffeNet [54]	TGRS2017
	GoogleNet [20]	TGRS2017
	VGG-VD-16 [20]	TGRS2017
	ResNet18 [10]	TNNLS2022
Multi-scale Network	EFPN-DSE-TDFF [30]	TGRS2021
	MSA-Network [20]	JSTARS2021
	MF^2^CNet [55]	TGRS2022
	MGSNet [56]	TGRS2023
	MBFANet [29]	GRSL2023
	CGINet [57]	TGARS2024
	Res2SE50 [58]	ICICML2024
	TAKD [59]	TCSVT2024
	SAF-Net [60]	GRSL2024
Transformer-Related	ViT-B32 [20]	ICLR2021
	Swin-T [61]	ICCV2021
	PVT-V2-B0 [20]	CVM2022
	HHTL [58]	JSTARS2022
	SCViT [15]	TGARS2022
	EMTCAL [20]	TGARS2022
	LTNet [62]	RS2023

**Table 2:** Comparison of OA and Standard Deviations (%) of Comparison of State-of-Art Method on UCM Dataset.

Methods	UCM	
	Tr = 50%	Tr = 80%
CaffeNet	93.98 ± 0.67	95.02 ± 0.81
GoogleNet	92.70 ± 0.60	94.31 ± 0.89
VGG-VD-16	94.14 ± 0.69	95.21 ± 0.20
ResNet18	97.43 ± 0.19	99.05 ± 0.25
EFPN-DSE-TDFF	96.19 ± 0.19	99.14 ± 0.22
MSA-Network	97.80 ± 0.33	98.96 ± 0.21
MF^2^CNet	98.76 ± 0.18	99.52 ± 0.25
MGSNet	-	99.76 ± 0.14
MBFANet	-	99.66 ± 0.19
CGINet	-	99.84 ± 0.16
Res2SE50	-	-
TAKD (ResNet18)	97.10	98.42
SAF-Net	-	99.32 ± 0.16
ViT-B32	97.83 ± 0.16	98.95 ± 0.24
Swin-T	-	99.46 ± 0.11
PVT-V2-B0	97.94 ± 0.44	98.86 ± 0.38
HHTL	98.87 ± 0.28	99.48 ± 0.28
SCViT	98.90 ± 0.19	99.57 ± 0.31
EMTACL	98.67 ± 0.16	99.57 ± 0.28
LTNet	98.36 ± 0.25	-
HETMCL	99.14 ± 0.33	99.76 ± 0.16

**Table 3:** Comparison of OA and Standard Deviations (%) of Comparison of State-of-Art Method on AID Dataset.

Methods	AID	
	Tr = 20%	Tr = 50%
CaffeNet	86.86 ± 0.47	89.53 ± 0.31
GoogleNet	83.44 ± 0.40	89.64 ± 0.36
VGG-VD-16	86.59 ± 0.29	89.64 ± 0.36
ResNet18	93.36 ± 0.12	95.51 ± 0.31
EFPN-DSE-TDFF	94.02 ± 0.21	94.50 ± 0.30
MSA-Network	93.53 ± 0.30	96.01 ± 0.43
MF^2^CNet	95.54 ± 0.17	97.02 ± 0.28
MGSNet	95.46 ± 0.21	97.18 ± 0.16
MBFANet	93.98 ± 0.15	96.93 ± 0.16
CGINet	93.35 ± 0.15	97.10 ± 0.24
Res2SE50	94.09 ± 0.23	96.46 ± 0.19
TAKD(ResNet18)	92.20	95.35
SAF-Net	94.20± 0.12	96.72± 0.14
ViT-B32	93.74 ± 0.27	95.84 ± 0.29
Swin-T	94.56 ± 0.14	96.92 ± 0.12
PVT-V2-B0	93.52 ± 0.35	96.27 ± 0.14
HHTL	95.62 ± 0.13	96.88 ± 0.21
SCViT	95.56 ± 0.17	96.68 ± 0.16
EMTACL	94.69 ± 0.14	96.41 ± 0.23
LTNet	94.98 ± 0.25	-
HETMCL	95.91 ± 0.27	97.32 ± 0.10

**Table 4:** Comparison of OA and Standard Deviations (%) of Comparison of State-of-Art Method on NWPU Dataset.

Methods	NWPU	
	Tr = 10%	Tr = 20%
CaffeNet	-	-
GoogleNet	76.19 ± 0.38	78.48 ± 0.26
VGG-VD-16	-	-
ResNet18	88.91 ± 0.23	91.77 ± 0.18
EFPN-DSE-TDFF	-	-
MSA-Network	90.38 ± 0.17	93.52 ± 0.21
MF2CNet	92.07 ± 0.22	93.85 ± 0.27
MGSNet	92.40 ± 0.16	94.57 ± 0.12
MBFANet	91.61 ± 0.14	94.01 ± 0.08
CGINet	92.28 ± 0.17	94.38 ± 0.13
Res2SE50	91.52 ± 0.43	94.13 ± 0.28
TAKD(ResNet18)	89.10	92.26
SAF-Net	90.94 ± 0.08	93.62 ± 0.10
ViT-B32	90.05 ± 0.29	92.61 ± 0.14
Swin-T	90.84 ± 0.09	93.18 ± 0.15
PVT-V2-B0	89.72 ± 0.16	92.95 ± 0.09
HHTL	92.07 ± 0.44	94.21 ± 0.09
SCViT	92.72 ± 0.04	94.66 ± 0.10
EMTACL	91.63 ± 0.19	93.65 ± 0.12
LTNet	92.21 ± 0.11	-
HETMCL	92.54 ± 0.18	95.02 ± 0.14

**Table 5:** Result of Ablation Experiments For AFFM.

Net	AID	
	Tr = 20%	Tr = 50%
0	95.01	96.98
1	95.33	97.12
2	95.91	97.32

**Table 6:** Result of Ablation Experiments For HFIE.

Net	AID	
	Tr = 20%	Tr = 50%
0	94.87	96.70
1	94.98	96.86
2	95.54	97.22
3	95.91	97.32

**Table 7:** Result of Ablation Experiments For DFE.

Net	AID	
	Tr = 20%	Tr = 50%
0	95.61	97.27
1	95.41	97.18
2	95.91	97.32

**Table 8:** Result of Ablation Experiments For MCAA.

Net	AID	
	Tr = 20%	Tr = 50%
0	95.56	96.82
1	95.76	97.28
2	95.91	97.32

**Table 9:** Gflops And Oa Of HETMCLs With Different Transformer Architectures.

Net	GFLOPs	Para(M)	Inference Time (ms)	FPS	Tr = 20%	Tr = 50%
with ViT	11.20	19.76	19.61	50.98	95.01	96.82
with PVT	10.82	19.16	19.56	51.14	95.13	96.96
with Swin	10.36	18.48	19.34	51.72	95.19	97.06
with CMT	10.64	18.88	19.49	51.31	95.17	97.03
with HFIE	12.28	20.08	19.91	50.23	95.91	97.32

**Table 10:** Quantitative Analysis Results:.

Model Configuration	Power_low_ (%)	Power_low_ (%)	Edge Preservation Index (EPI)
Low-Frequency Only	83.2 ± 1.7	16.8 ± 1.2	0.31 ± 0.04
Full HFIE	68.5 ± 2.3	31.5 ± 1.9	0.58 ± 0.06

**Table 11:** OA and Kappa of Three Datasets.

Datasets	OA	Kappa
UCM (5:5)	0.9914	0.9900
UCM (8:2)	0.9976	0.9920
AID (2:8)	0.9591	0.9575
AID (5:5)	0.9732	0.9725
NWPU (1:9)	0.9254	0.9210
NWPU (2:8)	0.9502	0.9473

## Data Availability

The data that support the findings of this study are available from the corresponding author upon reasonable request.
